# Efficacy of IAPP suppression in mouse and human islets by GLP-1 analogue conjugated antisense oligonucleotide

**DOI:** 10.3389/fmolb.2023.1096286

**Published:** 2023-02-06

**Authors:** Tatyana Gurlo, Thazha P. Prakash, Zhongying Wang, Maani Archang, Lina Pei, Madeline Rosenberger, Elaine Pirie, Richard G. Lee, Peter C. Butler

**Affiliations:** ^1^ Larry L. Hillblom Islet Research Center, David Geffen School of Medicine, University of California, Los Angeles, Los Angeles, CA, United States; ^2^ IONIS Pharmaceuticals, Carlsbad, CA, United States

**Keywords:** islet amyloid polypeptide (IAPP), antisense oligonucleotide (ASO), glucagon-like peptide 1 (GLP-1), islet, diabetes

## Abstract

Insulin resistance is the major risk factor for Type 2 diabetes (T2D). In vulnerable individuals, insulin resistance induces a progressive loss of insulin secretion with islet pathology revealing a partial deficit of beta cells and islet amyloid derived from islet amyloid polypeptide (IAPP). IAPP is co-expressed and secreted with insulin by beta cells, expression of both proteins being upregulated in response to insulin resistance. If IAPP expression exceeds the threshold for clearance of misfolded proteins, beta cell failure occurs exacerbated by the action of IAPP toxicity to compromise the autophagy lysosomal pathway. We postulated that suppression of IAPP expression by an IAPP antisense oligonucleotide delivered to beta cells by the GLP-1 agonist exenatide (eGLP1-IAPP-ASO) is a potential disease modifying therapy for T2D. While eGLP1-IAPP-ASO suppressed mouse IAPP and transgenic human IAPP expression in mouse islets, it had no discernable effects on IAPP expression in human islets under the conditions studied. Suppression of transgenic human IAPP expression in mouse islets attenuated disruption of the autophagy lysosomal pathway in beta cells, supporting the potential of this strategy.

## Introduction

Type 2 diabetes (T2D) is an increasingly common metabolic disorder attributed to impaired insulin secretion in the setting of relative insulin resistance. Insulin resistance is a prominent risk factor for T2D in those who are predisposed, for example due to obesity (Abdullah et al., 2010), pregnancy (Johns et al., 2018; Salazar-Petres and Sferruzzi-Perri, 2022), treatment with glucocorticoids (reviewed in (Beaupere et al., 2021)) or hepatic cirrhosis (Lee et al., 2019).

Most individuals adapt to insulin resistance by an appropriate increase in insulin secretion. However, in a vulnerable subset of the population insulin secretion fails to sufficiently increase in response to insulin resistance leading to T2D. While there are many potential causes of the initial failure of beta cell adaptation to increased demand, there is accumulating evidence that protein misfolding contributes to the progressive decline in beta cell function after onset of T2D (Mukherjee et al., 2015).

The islets of Langerhans in T2D have the classical hallmarks of a protein misfolding disorder with amyloid deposits of a locally expressed protein, islet amyloid polypeptide (IAPP). IAPP is co-expressed and co-secreted with insulin by pancreatic beta cells, and its relative expression increases under conditions of insulin resistance (Butler et al., 1990; Koranyi et al., 1992). The amyloidogenicity of IAPP predicts species vulnerability to T2D (Costes et al., 2013). Islet pathology and metabolic changes comparable to T2D can be reproduced in rodents with transgenic expression of human IAPP (hIAPP) (Matveyenko and Butler, 2006). The induction of diabetes in these models is increased in relation to the expression of hIAPP, whether by gene dose or induction of insulin resistance. These models were among the first to establish that the toxicity of amyloidogenic proteins is mediated by intracellular membrane permeant oligomers rather than the extracellular amyloid that had been thought to be responsible and predicated the failed trials of antibodies directed at extracellular amyloid (Lin et al., 2007). Further, we and others were able to demonstrate that IAPP misfolded protein stress compounds beta cell toxicity by disrupting the key defense mechanisms that clear misfolded proteins such as the autophagy lysosomal pathway (Rivera et al., 2014; Shigihara et al., 2014; Kim et al., 2014) Taken together these findings imply that a therapeutic strategy that decreases IAPP expression to suppress toxic oligomer formation might be a disease modifying therapy in T2D.

In a recent study it was shown that use of a GLP-1 analog exenatide (eGLP1) was able to deliver antisense oligonucleotides (ASO) to pancreatic beta cells in mice and suppress expression of rodent IAPP (Knerr et al., 2021). We sought to extend that work to establish if the same approach could suppress expression of human IAPP in beta cells as a potential therapeutic strategy in T2D.

We addressed this question using a beta cell specific human hIAPP transgenic mouse model that develops diabetes attributable intracellular membrane permeant hIAPP oligomers reminiscent of those in beta cells in humans with T2D (Gurlo et al., 2010).

## Methods

### Mouse models

Animal studies were approved by the UCLA Office of Animal Research Oversight. The transgenic mouse strain expressing hIAPP (hTG), available from Jackson Laboratory, Bar Harbor, ME, United States: IMSR cat. No. JAX:008232, RRID:IMSR_JAX:008232) is described elsewhere (Janson et al., 1996). hTG males develop diabetes between 9 and 12 weeks of age (Gurlo et al., 2016). The wild-type FVB mice (WT; IMSR cat. No. CRL:207, RRID:IMSR_CRL:207) were originally purchased from Charles Rivers Laboratory (Wilmington, MA, United States). All mice were bred at UCLA, and were maintained on a 12-h day/night rhythm with Harlan Teklad Rodent Diet 8604 (Placentia, CA, United States) and water *ad libitum*. Heterozygous hIAPP transgenic mice (hTGhet) were obtained by cross-breeding of hTG male to FVB female. Diabetes was monitored by measurement of blood glucose in a tail-tip blood sample with a FreeStyle Freedom Lite Glucometer (Abbott, Alameda, CA, United States). The number of mice used for each experiment is provided in the figure legends.

### Human islets

Human pancreatic islets (RRID numbers SAMN15656747, SAMN28501433, SAMN11982795) and/or other resources were provided by the NIDDK-funded Integrated Islet Distribution Program (IIDP) (RRID:SCR_014387) at City of Hope, NIH Grant #2UC4DK098085.

The donors (*n* = 3, aged 24, 48 and 54 years) were non-diabetic. Islets were cultured in RPMI 1640 medium (5.5 mmol/l glucose) containing 100 IU/ml penicillin, 100 μg/ml streptomycin and 10% (vol./vol.) fetal bovine serum (FBS, Atlanta Biologicals, Flowery Branch, GA, United States) before transplantation or *in vitro* experiments.

### Islet transplantation

The immunodeficient scid-beige mice (Model#CBSCBG-M; C.B-*Igh*-1b/GbmsTac-*Prkdc* ^*scid*^ *-Lyst* ^*bg*^ N7) used as a recipient of human islet transplant were purchased from Taconic (Rensselaer, NY, United States), and were housed in a pathogen free facility before and after human islet transplantation. Islet transplantation was performed as described elsewhere (Pagliuca et al., 2014). 10–17 weeks old mice received 1,500–2,000 IEQ per mouse. 4–8 weeks later, blood was collected to confirm the presence of human insulin in the blood stream using human insulin ELISA (80-INSHU-E01, ALPCO, Salem, NH, United States) before initiation of treatment with ASO. Successful engraftment of human islets was affirmed by the presence of plasma human insulin in all 12 transplanted mice but not in non-transplanted controls. Fasting plasma insulin ranged from 4.7 to 31.0 μIU/ml, sensitivity of the assay 0.135 μIU/ml.

### ASO treatment of mice

ASO synthesis was carried out as previously described (Ammala et al., 2018). hTG mice were treated with ASO weekly in the morning in non-fasting state starting at 6–7 weeks of age. Stock ASO was diluted in saline, and 150–250 μl per mouse were delivered s.c. (over the shoulders, into the loose skin over the neck) at 1 mg/kg or 5 mg/kg dose. The scid-beige mice transplanted with human islets received three weekly injections of ASO at 5 mg/kg. The concentration of ASO in the working solution was monitored using Nanodrop One (Thermo Scientific), and the injection volume was calculated based on the non-fasting body weight measurement. For short-term experiments, hTG mice received 2 injections on day 1, and 4, and islets were isolated on day 7. In long-term experiments, mice were treated for 12 weeks, islets were collected 3–5 days after the last ASO injection. Diabetes was monitored by measuring blood glucose at fasting state 3–5 days post ASO injection. Mouse was considered diabetic when fasting blood glucose level reached 125 mg/dl.

### Pancreas harvesting, islet isolation and lysate preparation

Islets were isolated by collagenase digestion as previously described (Rivera et al., 2014). Islets were manually picked, washed twice with ice-cold PBS, and frozen either in NP40 lysis buffer [20 mM Tris-HCl, 150 mM NaCl, 2 mM MgCl2, 0.5% NP-40, 1 mM DTT, 5 mM NaF, 1 mM Na3VO4, and protease inhibitor cocktail from Sigma (Cat#P2714, Sigma-Aldrich, St. Louis, MO, United States)] or in RLT buffer from Qiagen RNeasy MiniKit (Qiagen, Germantown, MD, United States) supplemented with bME (Sigma-Aldrich) at −80°C until use for protein and RNA analysis, respectively. To preserve a sample of pancreas for the immunohistochemical analysis, we ligated a small portion of the pancreatic tail with the surgical suture before perfusion to prevent the entrance of the perfusion solution, dissected it from the inflated pancreas, fixed with 4% PFA overnight, and embedded in paraffin.

Kidneys from the scid-beige mice transplanted with human islets were dissected before islet isolation from the pancreas. Left kidneys with transplanted islets were removed, the area containing islets was dissected, weighted, and homogenized in the ice-cold RLT buffer from Qiagen RNeasy MiniKit (Qiagen, Germantown, MD, United States) supplemented with bME (Sigma-Aldrich), and short-term stored frozen at −80°C before RNA extraction. Portions of the right kidneys were used as negative controls.

### ASO treatment of islets

For *in vitro* testing of ASO activity, islets were isolated from WT mice or prediabetic hTG mice, cultured for 24–48 h in RPMI 1640 medium supplemented with 100 IU/ml penicillin, 100 μg/ml streptomycin and 10% FBS (vol./vol.) at 37°C, in a humidified 5% CO_2_ atmosphere to allow recovery. Then islets were plated into suspension 6 well plates at 50–70 per well in 1.5 ml of TCM, and ASO diluted in saline was added at 500 nM final concentration. 24 h later, islets were picked and lysed in 350 ul of Qiagen RLT buffer supplemented with bME and frozen until further use for RNA analysis. Human islets were treated with 500 nM or 1 μM ASO. As controls, GLP-1 or eGLP1-Control-ASO were used as described in figure legends.

### RNA isolation and analysis

RNA was isolated using Qiagen RNA Mini kit according to the manufacturer instructions. Single stranded cDNA was prepared using Superscript III First-Strand Synthesis System Kit from Invitrogen. Real time quantitative PCR was performed using FAST SYBRGreen master mix and ABI7900HT or QuantStudio 6 Flex equipments from Applied Biosystems (Foster City, CA, United States). The following mouse primers were used *Iapp*—gatgtgcatctccaaactgc and ttg​tcc​atc​tga​ggg​ttg​ct, *Malat1*—tgggttagagaaggcgtgtactg and tca​gcg​gca​act​ggg​aaa, *Hprt1*—ctcctcagaccgctttttgc and taa​cct​ggt​tca​tca​tcg​cta​atc; *Hprt1* was used as a housekeeping gene. Human primers were *IAPP*- cca​ttg​aaa​gtc​atc​agg​tgg​a and cca​cgt​tgg​tag​atg​aga​gaa​tg. *MALAT1*—aaagcaaggtctccccacaag and tga​agg​gtc​tgt​gct​aga​tca​aaa; *HPRT1*—gaccagtcaacaggggacat and gtg​tca​att​ata​tct​tcc​aca​atc​aag; *HPRT1* was used as a housekeeping gene.

### Western blotting

Islet samples were defrosted on ice, sonicated and spun at 10,000 g at 4°C for 10 min. Protein concentration in the lysate supernatant was measured using DC protein assay (Bio-Rad, Hercules, CA, United States). Proteins (20 μg per lane) were separated on 4%–12% BisTris NuPAGE gels (Invitrogen, Carlsbad, CA, United States) and blotted onto a PVDF membrane (Pall, Ann Arbor, MI, United States). Membranes were blocked in 5% milk (BioRad), incubated over night with primary antibodies diluted in the antibody buffer (TBS, 0.1% Tween 20, 5% BSA) and then probed with horseradish peroxidase-conjugated secondary antibodies from Life Technologies. Proteins were visualized using ECL reagents from BioRad or Millipore. Protein expression levels were quantified using Labworks software (UVP, Upland, CA, United States).

### Antibodies

We used rabbit anti-GAPDH (Cell Signaling Technology Cat# 2118, RRID:AB_561053, 1:1000 for WB) and anti-IAPP (Peninsula Laboratories Cat# T-4157.0400, RRID:AB_518725, 1:1000 for WB); guinea pig anti-insulin (Abcam Cat# ab7842, RRID:AB_306130, 1:300 for IF) and anti-p62 (Progen Cat# GP62-C, RRID:AB_2687531, 1:400 for IF), mouse anti-polyubiquitin K63-linkage-specific (clone HWA4C4, Enzo Life Sciences Cat# BML-PW0600, RRID:AB_10540645, 1:100 for IF). Secondary antibodies for immunofluorescence staining F(ab’)2 conjugates with Cy3 or FITC obtained from The Jackson Laboratories (West Grove, PA, United States) were used at dilution of 1:200, and for western blotting HRP conjugated goat anti-rabbit IgG antibody from Invitrogen was used at 1:3000 dilution.

### Immunostaining

Staining was performed using 4-μm sections following the protocol described in (Huang et al., 2010). The frequency of beta cell containing P62 and polyubiquitinated proteins inclusions were assessed in the adjacent sections co-stained for P62 and insulin or K63 polyubiquitin and insulin, and were mounted with Vectashield with DAPI (Vector Laboratories cat. No. H-1200, RRID:AB 2336790). All islets on the section were viewed using a Leica DM6000 fluorescent microscope with a ×20 objective and imaged using OpenLab 5.5 software (Improvision). Six to thirty islets per case were imaged; the number of insulin positive beta cells counted were 1,626 and 1,498 per control and hIAPP ASO treatment in 1 mg/kg groups, and counted being 2,187 and 6,838 in 5 mg/kg groups respectively. Image analysis was performed blindly by two independent investigators (TG and ZW). The frequency of beta cells with impaired autophagosome/lysosome mediated protein degradation was expressed as percentage of insulin positive cells containing P62 inclusions.

### Statistical analysis

Results are expressed as the mean ± SEM when appropriate. Statistical analyses were carried out by Student’s t-test GraphPad software. *p* <.05 was taken as evidence of statistical significance; *p <.05; ***p <.001.

## Results

### Efficacy and selectivity of suppression of human IAPP expression by an IAPP antisense oligonucleotide delivered to beta cells by a GLP-1 analog

**FIGURE 1:**
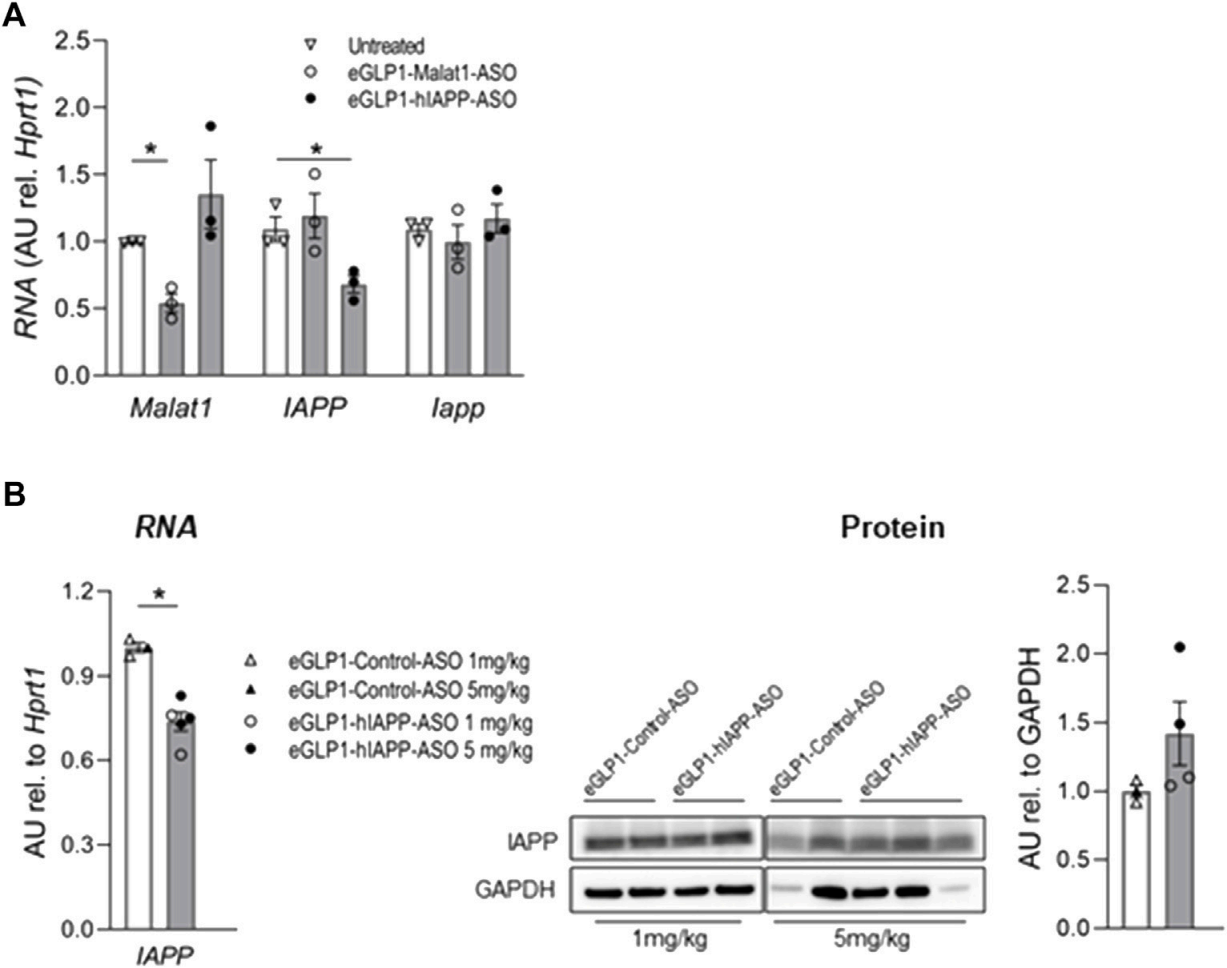
Evaluation of eGLP1-hIAPP-ASO in mouse islets *in vitro* and *in vivo*. **(A)** eGLP1-hIAPP-ASO (500 nM) specifically decreases expression hIAPP in hTG islets after *in vitro* 24 h treatment; representative experiment performed in triplicates; eGLP1-Malat1-ASO was used as positive control. **(B)** Short-term treatment of hTG mice with eGLP1-hIAPP-ASO (filled bars) resulted in the decrease in hIAPP RNA without effect on protein level of monomeric IAPP compared to mice treated with eGLP1-Control-ASO (eGLP1 peptide conjugated to an ASO with a scrambled nucleotide sequence; open bars). Mice received either 1 mg/kg (open symbols) or 5 mg/kg (filled symbols) of ASO on day 0 and 4, and islets were isolated on day 7. Only samples with strong GAPDH signal were included in the western blot quantification. Data is mean ± SEM; * - *p* <0.05, two-tailed non-paired Student’s test.

In order to evaluate the potential for an antisense oligonucleotide approach to suppress expression of human IAPP we first incubated islets isolated from hTG mice for 24 h with 500 nM eGLP1-hIAPP-ASO (Figure 1A). By this approach hIAPP RNA was suppressed by 32.7 ± 6.3% (*p* <0.01) but mouse IAPP RNA was not altered. Suppression of *Malat1* has previously been used as a positive control for ASO targeted activity, and application 500 nM eGLP1-Malat1-ASO suppressed islet *Malat1* RNA expression by 59.1 ± 6.3% (*p* <0.01).

In order to extend these findings *in vivo*, we injected hTG mice with eGLP1-hIAPP-ASO versus eGLP1-control-ASO at two doses (1 mg/kg or 5 mg/kg) on day 0 and 4, and isolated islets on day 7. While hIAPP RNA was decreased in this 7-day study (Figure 1B), we did not observe any change in islet monomeric IAPP protein expression.

### Impact of human IAPP ASO treatment on development of diabetes in human IAPP transgenic mice

**FIGURE 2:**
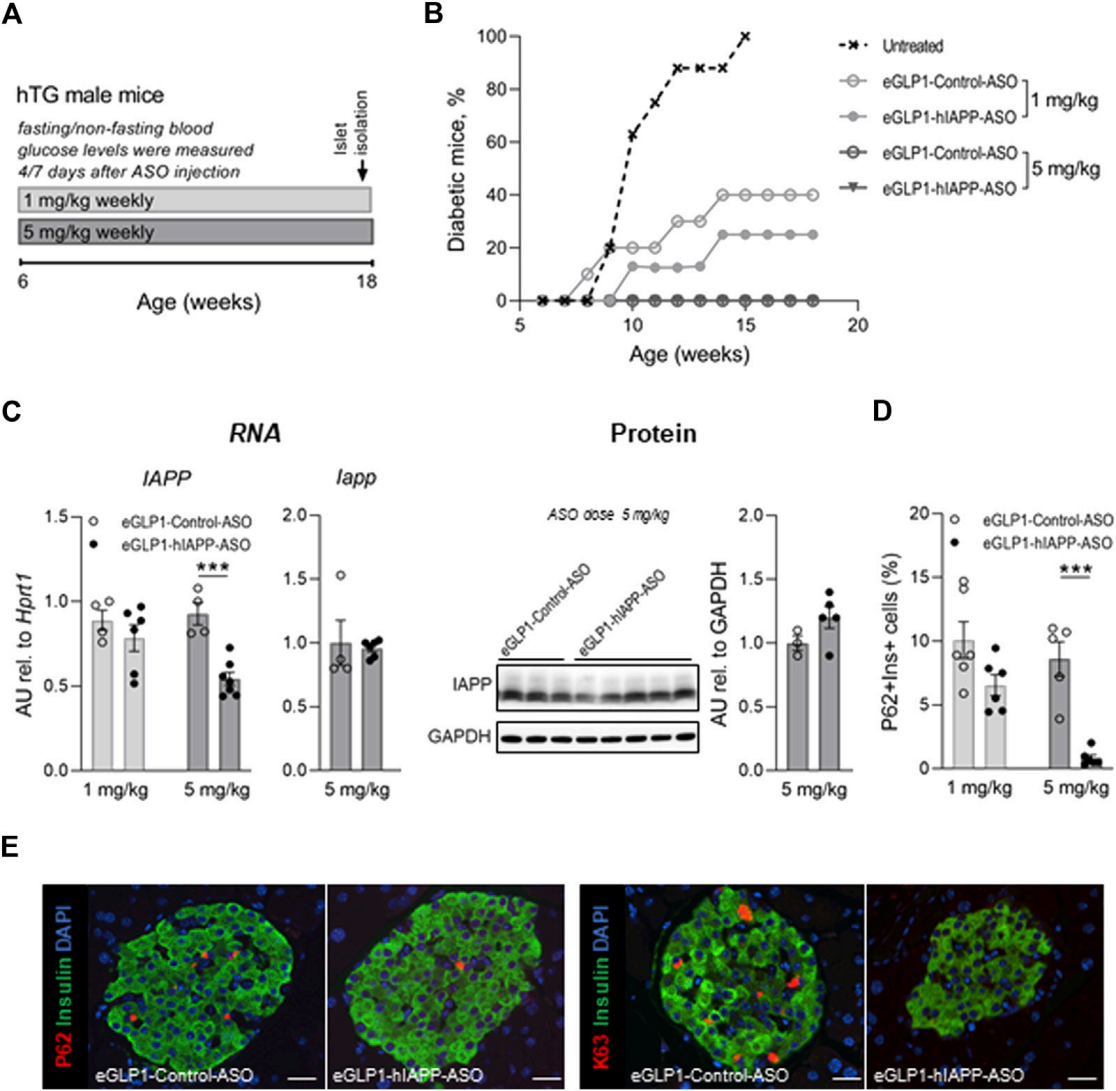
Effect of treatment with eGLP1-hIAPP-ASO on diabetes development and autophagy/lysosome dependent pathway of protein degradation in beta cells. **(A)** Scheme of the experiment. Mice were treated weekly with eGLP1-hIAPP-ASO or with eGLP1-Control-ASO (eGLP1 peptide conjugated to an ASO with a scrambled nucleotide sequence). **(B)** Diabetes development (mouse was considered to be diabetic if blood glucose levels measured after overnight was >125 mg/dl or non-fasting morning glucose aver 250 mg/dl). **(C)** Islet hIAPP RNA levels were decreased in dose dependent manner without noticeable effect on levels of the monomeric hIAPP protein relative to e-GLP1-Control-ASO treated mice. **(D)** The frequency of beta cells containing P62 positive inclusions decreased in eGLP1-hIAPP-ASO treated mice in a dose dependent manner. **(E)** Representative images of islets stained for P62 and K63 polyubiquitinated proteins destined for the autophagosome/lysosome mediated degradation (scale bar 25 mm). **(C–E)** Data are from non-diabetic mice only. Data are presented as the mean ± SEM; n = 5–7 in (b), *n* = 4–6 in (c); *n* = 5–6 in (f); *** - *p* <0.001, two-tailed non-paired Student’s test; hTG—human IAPP transgenic.

We next sought to establish if the trajectory of diabetes development could be attenuated in the hTG mouse model of T2D by beta cell targeted hIAPP ASO treatment. We studied five groups of hTG mice from age 6–18 weeks to encompass the period of diabetes onset in this mouse model. In four groups of mice we administered weekly doses of either eGLP1-hIAPP-ASO (1 mg/kg or 5 mg/kg) or eGLP1-Control-ASO (1 mg/kg or 5 mg/kg), the latter 2 groups to control for the actions of GLP-1 (Figure 2A). The 5th group received no treatment and as expected 95% developed diabetes by 15 weeks of age. Consistent with prior reports (Moffett et al., 2015) (Dhanesha et al., 2013), GLP-1 (eGLP1-Control-ASO) at 1 mg/kg delayed diabetes onset with ∼60% remaining non-diabetic by 18 weeks of age. The addition of hIAPP ASO to GLP-1 (eGLP1-hIAPP-ASO) at the same dose further delayed the onset of diabetes (by an additional 15% at 18 weeks of age). Using the higher dose of 5 mg/kg of both eGLP1-hIAPP-ASO and eGLP1-Control-ASO fully suppressed development of diabetes (0% diabetes at 18 weeks; Figure 2B).

There was no detectable difference in hIAPP RNA in islets from the eGLP1-hIAPP-ASO and eGLP1-control-ASO mice treated at 1 mg/kg but an ∼50% suppression of hIAPP RNA by the eGLP1-hIAPP-ASO when the ASOs were administered at 5 mg/kg. There were no clear differences in monomeric IAPP protein (mouse plus human) in islet extracts between groups at either dose of ASO or from untreated mice (Figure 2C), likely due to the fact that the antibody used in this assay detects both mouse and human IAPP protein, while hIAPP ASO is only effective in reducing human IAPP. In addition, our hIAPP transgene is a cDNA construct and ASOs are known to work more efficiently on genomic transgenes, which may explain eGLP-1-hIAPP-ASO being less active in our model than other eGLP-1 conjugated ASOs previously reported in non-transgenic mice.

### Effect of hIAPP ASO on the autophagy/lysosomal pathway in beta cells of hIAPP transgenic mice

Compromised autophagy/lysosomal function is a well-established consequence of protein misfolding stress, including beta cells in humans with T2D (Rivera et al., 2011; Rivera et al., 2014; Mizukami et al., 2014). The p62 sequestome binds ubiquitinated aggregated misfolded proteins and delivers them into the autophagy lysosomal pathway of protein degradation (Knaevelsrud and Simonsen, 2010). When this pathway is compromised, p62 accumulates in detectable punctate sequestomes characteristic of beta cells in T2D (Figure 2D; Supplementary Figure S1). Despite the beneficial effects of GLP-1 signaling on delaying diabetes onset, there was still clear evidence of autophagy lysosomal dysfunction in beta cells from hTG mice treated with eGLP1-control-ASO at both 1 and 5 mg/kg (Figure 2E). In contrast, formation of p62 sequestomes was partially attenuated by treatment with hIAPP-ASO at 1 mg/kg and absent at 5 mg/kg.

### The efficacy of eGLP1 delivered ASOs in human beta cells is lower than in rodent beta cells

Targeted delivery of a therapy to pancreatic beta cells in humans is a challenge based on the small target cell mass (∼1 g in a 70–100 kg human). Since GLP-1 binds with relative avidity to pancreatic beta cells, it was rationale to use a GLP-1 analog (eGLP1) to target ASO therapy to beta cells. As noted previously, (Knerr et al., 2021), this has been effective in rodents but there is increasing evidence that rodent and human islets have many differences (Cabrera et al., 2006; Campbell et al., 2020; de Souza et al., 2020). Therefore, we next sought to examine the efficacy of the eGLP1-hIAPP-ASO to suppress IAPP expression in human islets.

**FIGURE 3:**
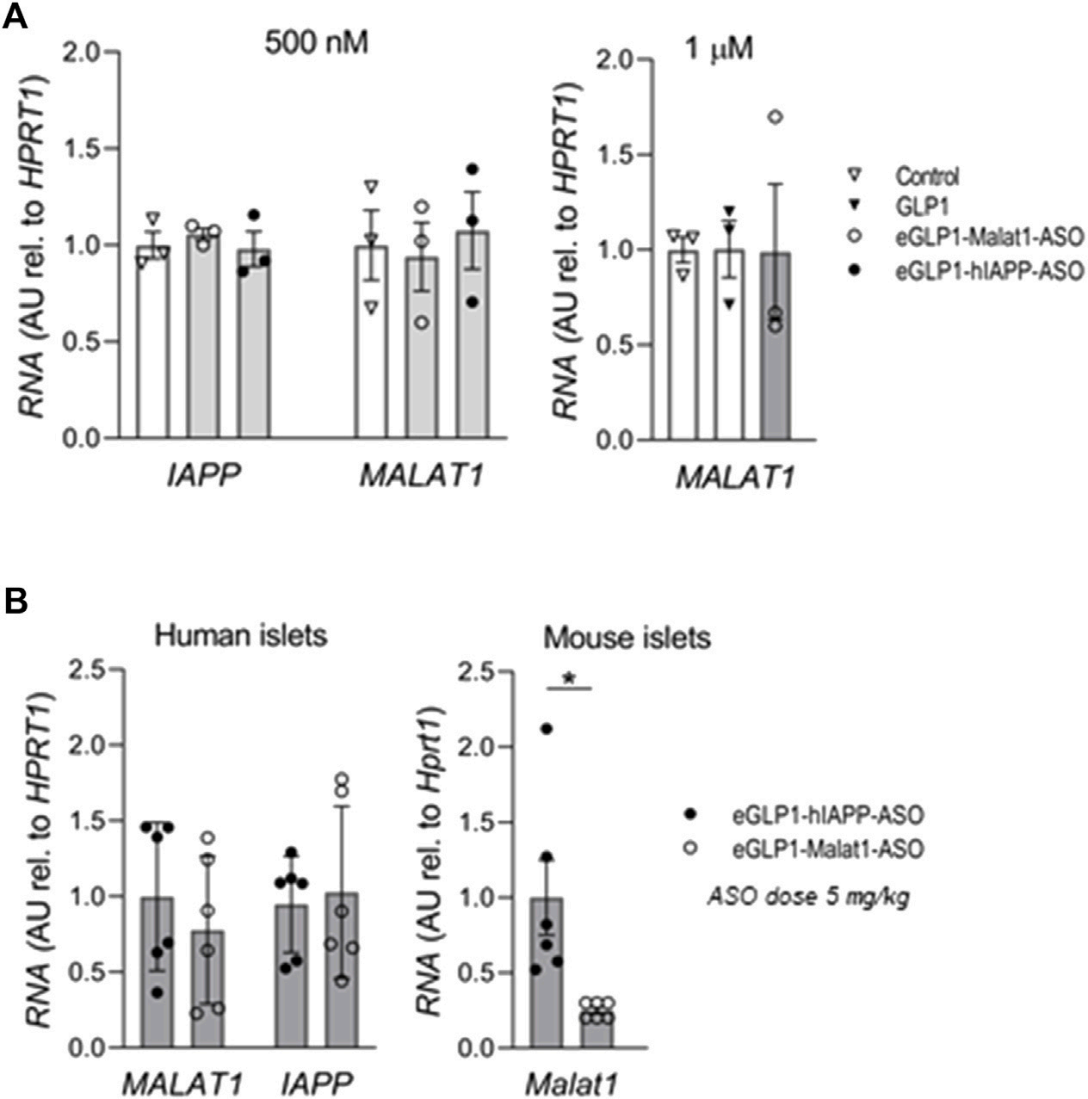
The efficiency of eGLP1-ASO is lower in human islets than in mouse islets. **(A)** Human islets were cultured for 24 h in the presence of 500 nM or 1 μM of eGLP1-Malat1-ASO, eGLP1-hIAPP-ASO or GLP-1 in two independent experiments performed in triplicates. **(B)** Scid-Beige mice were transplanted with human islets from a non-diabetic donor and received three weekly injections of ASO at 5 mg/kg. At the end of the experiment, RNA was extracted from the kidney transplants and from the isolated islets from mouse pancreas. Data is mean ± SEM; *p <0.05.

In contrast to mouse islets, we were unable to demonstrate any suppression of *IAPP* or *MALAT1* in human islets by eGLP1 targeted human IAPP or MALAT1 ASOs *in vitro* (Figure 3A) or after transplant of human islets in Scid-Beige mice *in vivo* (Figure 3B). In the same *in vivo* mouse experiment, we reconfirmed suppression of *Malat1* by eGLP1-Malat1-ASO in the mouse islets.

## Discussion

Our primary goal in these studies was to address the hypothesis that an ASO targeting IAPP expression by beta cells could be used as a potential disease modifying therapy in T2D.

We reproduced prior findings (Ammala et al., 2018; Knerr et al., 2021; Yong et al., 2021) that an ASO conjugated to eGLP1 decreased expression of the ASO target in mouse beta cells *in vitro* and *in vivo*. Moreover, eGLP1 successfully delivered a hIAPP ASO to beta cells in islets from hTG mice, delaying diabetes and preserving the beta cell autophagy lysosomal pathway characteristic of misfolded protein stress.

While the same eGLP1 conjugated hIAPP ASO had no discernable effect on hIAPP RNA levels in human islets *in vitro or in vivo* when delivered at comparable doses, there are several possible explanations for this.

Isolation of human islets from brain dead human organ donors may disrupt access of GLP-1 to beta cell GLP-1 receptors *in vitro* or after transplant in a manner that does not occur following isolation of mouse islets rendering the GLP-1 receptors non-functional. Another explanation is the relative expression of GLP-1 and glucagon by alpha cells in the two species. Alpha cells in mice express predominantly glucagon but human alpha cells express both glucagon and GLP-1 at comparable levels (Campbell et al., 2020) to the extent that beta cell GLP-1 receptors in human islets are largely saturated with locally released GLP-1 at basal glucose values (de Souza et al., 2020). The same investigators showed that despite this saturation of GLP-1 receptors at basal glucose, they were able to elicit a GLP-1 enhanced glucose induced insulin response, likely because at high glucose alpha cell secretion is suppressed. Other explanations include possible differences in endocytosis of G-coupled receptor ligands in human versus mouse beta cells, and access of ASOs taken up at the cell membrane to the perinuclear region to limit protein expression through a cell type packed with membrane compartments and secretory vesicles.

The present studies in hTG mice did support the potential benefit that might be accomplished by suppression of hIAPP expression. Partial suppression of hIAPP RNA expression by the hIAPP-ASO in hTG mice delayed diabetes onset and preserved the autophagy lysosomal pathway that is critical in long lived cells with a high protein synthesis work load such as beta cells (Rivera et al., 2014). This beneficial effect was to some extent masked by the potent effect of the GLP-1 agonist eGLP1 to delay diabetes in hTG mice.

Of note, while GLP-1 agonist treatment delays diabetes in numerous rodent models of diabetes, this benefit does not extend to humans (Young et al., 1999; Moffett et al., 2015; Fan et al., 2018; Consortium, 2019). This species variance is likely due to the properties of GLP-1 agonist therapy to promote beta cell replication and suppress beta cell apoptosis. Beta cells in rodent models of diabetes retain a capacity for beta cell replication and typically have a high rate of beta cell apoptosis (Moffett et al., 2015; Hao et al., 2017). In contrast, beta cells in adult humans have minimal capacity for beta cell replication (Butler et al., 2007; Menge et al., 2008; Gregg et al., 2012; Dai et al., 2017). Also, in the setting of misfolded protein stress or other toxicity, beta cells in humans have a low frequency of apoptosis due to upregulation of potent pro-survival signaling at the expense of beta cell function (Montemurro et al., 2019).

In summary, while we reproduced the successful suppression of IAPP in mouse beta cells *in vitro* and *in vivo* by use of eGLP1, we were unable to extend this finding to human beta cells *in vitro* or *in vivo* after transplant in mice under the conditions of the present study. Suppression of hIAPP by the ASO approach in transgenic mice shows promise of this potential therapy by preserving the autophagy lysosomal pathway known to be critical in defense against protein misfolding diseases such as T2D. The approach could offer a disease modifying therapy for T2D.

## Data Availability

The raw data supporting the conclusion of this article will be made available by the authors, without undue reservation.
