# Supplementary material for: Efficacy of IAPP suppression in mouse and human islets by GLP-1 analogue conjugated antisense oligonucleotide
Source: Front Mol Biosci. 2023 Feb 6;10:1096286. doi: 10.3389/fmolb.2023.1096286 (PMC9939749; doi:10.3389/fmolb.2023.1096286)
Supplement: Supplementary file 1 [file Image1.pdf]

Supplementary Material

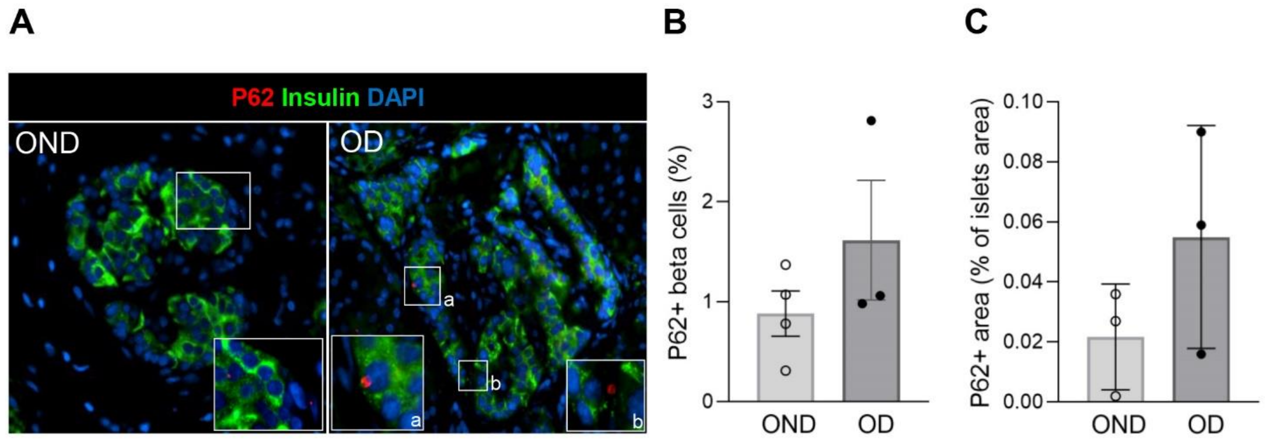

Supplementary Figure 1. P62 inclusions in human pancreatic islets. **(A)** Representative images of islets stained for P62 (marker for polyubiquitinated proteins destined for the autophagosome/lysosome mediated degradation) in islets from obese nondiabetic (OND) and obese subjects with type 2 diabetes (OD). The P62 protein positive inclusions appear to be more frequent in beta cells of OD subjects **(B)**, and occupy a larger area of the islets compared to OND subjects **(C)**. Data is Mean ± SEM, n=3-4 cases per group.
